# An exploration of the experiences of GP registrar supervisors in small rural communities: a qualitative study

**DOI:** 10.1186/s12913-020-05697-2

**Published:** 2020-09-05

**Authors:** Danielle Couch, Belinda O’Sullivan, Deborah Russell, Matthew McGrail, Glen Wallace, Michael Bentley

**Affiliations:** 1 School of Rural Health, Monash University, 26 Mercy St, Bendigo, VIC 3550 Australia; 2 Rural Clinical School, The University of Queensland, Toowoomba, Australia; 3 Menzies School of Health Research, Alice Springs, Northern Territory Australia; 4 General Practice Supervisors Australia, Bendigo, Australia; 5 General Practice Training Tasmania, Hobart, Australia

**Keywords:** General practice, Rural health, Registrar supervision, General practitioner training, business models

## Abstract

**Background:**

In Australia registrar training to become a general practitioner (GP) involves three to four years of supervised learning with at least 50% of GP registrars training wholly in rural areas. In particular rural over regional GP placements are important for developing future GPs with broader skills because the rural scope of practice is wider. Having enough GP supervisors in smaller rural communities is essential such training. We aimed to explore what makes rural GPs’ based outside of major regional centres, participate in supervising or not, their experiences of supervising, and impact of their practice context.

**Methods:**

Semi-structured interviews were undertaken with 25 GPs based in rural Tasmania (outside of major regions - Hobart and Launceston), in towns of < 25,000 population, to explore the GPs’ professional backgrounds, their experiences of supervising GP registrars, their practice context and their decisions about supervising GP registrars or not. Thematic analysis was undertaken; key ideas, concepts and experiences were identified and then reviewed and further refined to core themes.

**Results:**

Supervising was perceived to positively impact on quality of clinical care, reduce busy-ness and improve patient access to primary care. It was energising for GPs working in rural contexts. Rural GPs noted business factors impacted the decision to participate in supervision and the experience of participating: including uncertainty and discontinuity of registrar supply (rotational training systems), registrar competence and generating income.

**Conclusions:**

Supervising is strongly positive for rural GPs and related to job satisfaction but increasing supervision capacity in rural areas may depend on better policies to assure continuity of rural registrars as well as policies and systems that enable viable supervision models tailored to the context.

## Background

In Australia training to become a general practitioner (GP) as part of the Australian General Practice Training Program (AGPT) involves three to 4 years of college-accredited vocational training where the trainee is called a registrar. This training includes at least 50% of GP registrars doing their training wholly in rural (Modified Monash Model [MMM] 2–7) general practices [1]. Training takes place over consecutive placements each of 6 to 12 months duration, often in different practices [2]. Placements in rural locations are important for creating skilled rural GPs who are effective in rural practice environments and adept at working with rural population health issues [3]. GP registrars who train in rural settings are more likely to stay in rural practice [4]. However, rather than seeing all rural learning as the same, it is essential to understand the nuance of learning in smaller rural communities, over simply regional centres, so as to appraise the value of decentralising training and critique current AGPT training policy.

An adequate supply of rural GP supervisors is fundamental to enabling rural GP training in a practice. GP supervisors provide formative support and clinical exposure in a master/apprentice model between supervisors and registrars [5, 6]. Recent large increases in both the number of medical graduates from Australian universities and the number of general practice training positions [7], as well as targeted Australian Government policies aiming to grow and better distribute the general practice workforce in rural areas, creates an imperative for developing a supply of rural GP supervisors. Although this has occurred alongside other contemporary challenges to rural workforce distribution including a decrease in applicants for general practice training [8] and a decrease in entry into general practice [9, 10]. A 2019 national quantitative study identified that 58% of rural Australian GPs supervise registrars, with supervision of registrars associated with larger practice size, being Australian-trained and supervising interns and medical students [11]. Research from rural North West Queensland reveals that the distribution of rural GP supervisors was below the levels needed to address primary care need in some regions [12]. This was despite the explicit vision of the Regional Training Organisations (RTOs), (who deliver and monitor GP training in various geographic catchments nationally), to meet population needs by developing training placements and encouraging registrars to accept those placements in underserved areas.

Understanding the reasons GPs based in rural communities (outside of regional centres), supervise registrars, or not, can provide important evidence for targeting expanded participation, thereby potentially contributing to the agenda to decentralise training and contribute medical care to relatively underserved areas [6]. Of the scant literature on this topic, one study [13] interviewed rural GP supervisors in NSW and Victoria, not restricted to rural settings, finding that GPs are motivated to supervise by personal and professional factors, including professional development reasons, and because it was rewarding. A further study to inform growth of GP training in remote Queensland identified that supervising GP registrars in remote communities could be a way to enable unique high quality and team-based learning but practice busy-ness was a concern [14].

With this background in mind, this research aimed to better understand the supervision experiences of GPs in rural areas outside of regional centres, with a particular focus on describing their participation in supervising relative to context (personal, practice and community characteristics). We specifically aimed to explore this with GPs in smaller rural communities (< 25,000 population), where workforce distribution is most problematic, so as to inform decentralised GP training.

## Methods

Given our interest in the supervision experiences of GPs in rural areas, we used a phenomenological approach to investigate registrar supervision issues and experiences by exploring these from the perspective of those who have experienced them [15], in this case, GPs. Participants were recruited via email with a partnership with the single regional training organisation in Tasmania which manages GP registrar training. Tasmania, the setting for this study, is an Australian southern island state of less than one hour’s plane travel from mainland Australia. It has a population of approximately 524,000 [16] and is entirely rural according to the Australian Statistical Geography Standard classification.

Purposive sampling methods were used to seek differing context and experiences of supervision [17, 18], informed by the findings of a national quantitative study about the predictors of rural GPs supervising registrars [11]. Eligible participants were from rural towns all located outside of Hobart or Launceston (regional centres), and which had < 25,000 population. Participants included current formal (accredited) and informal (unaccredited), co- and main supervisors, GPs who used to supervise or co-supervise and GPs who were yet to take up supervision.

An interview guide was developed based on the research question, including prompts; this was informally piloted amongst the research team prior to being administered. Semi-structured interviews were conducted (October 2017–March 2018) via telephone or videoconferencing with only the interviewer and the participant present in each interview, and recorded. Interviews were of approximately 45 min duration. Interviews explored GP characteristics and practice arrangements and influences on decision making about whether to supervise GP registrars or not, including business considerations. An $A200 voucher was provided at completion of each interview. Ethics approval for the study was obtained from [Monash University 2017–10,808-13,857(14Sep2017)].

Two authors (BO’S and DR), who conceptualised this research and had a strong background in the broad issues and context related to practises of the rural medical workforce, undertook the interviews, documented and shared field notes following each interview to assist cross-learning, and met frequently to discuss developing themes. These authors, given their conceptualisation of the research topic, (they had noted this was a gap in the evidence base in their field), were motivated to hear the perspectives of the GPs in this study without any particular bias. Based on their strong history researching rural medical workforce issues, they were most able to interpret the GP narrative in real-time and prompt for further reflection equally across the participant interviews. A third author (DC) then led the thematic analysis using NVivo, reading the transcripts and recording ideas and thoughts, which were then discussed with the two interviewers (BO’S and DR). The transcripts were re-read, and further coded [19]. Regular meetings were held with the data analysis team to discuss and further refine coding; review and analysis continued and the codes were reduced to a number of core themes [20].

## Results

To aid data interpretation responses were described as follows: one to six participants as ‘few’, seven to 12 as ‘some’, 13–18 as ‘many’ and 19–25 as ‘most’. Key themes identified included supervision participation related to quality of clinical care, busy-ness of the practice, patient access and energising rural GPs. The final theme, concerning business factors, was the strongest.

### Characteristics of the sample and supervision participation

Thirty-one GPs indicated their interest in participating in the study, of whom 25 GPs were interviewed. Of the GPs not interviewed, one was away travelling, one did not meet the eligibility criteria, and remaining interested GPs were not eligible due to capped funding for the vouchers. Table 1 describes the participating GPs’ characteristics.

**Table 1:** Participant and practice characteristics

Participant characteristics	N
Gender	
Female	15
Male	10
Practice location ASGS-RA^a^ classification	
Inner regional	8
Outer regional	16
Remote	1
Practice size by FTE ^b^	
Less than or equal to 5 FTE	13
More than 5 FTE	12
Practice distance to nearest hospital	
<25kms	22
25-50kms	3
Practice distance to nearest large hospital ^c^	
<25kms	14
25-50kms	3
51-100kms	6
101 - 150kms	1
151-200kms	1
Practice supervises medical students	
Yes	20
No	4
Unknown	1

^a^*ASGS-RA* Australian Statistical Geography Standard-Remoteness Area

^*b*^ *FTE* Full-time equivalent

^c^ Royal Hobart Hospital, Launceston General Hospital, North West Regional Hospital and Mersey Community Hospital

All participants except one were either currently supervising in some manner, or wanted to return to or take up supervision if the conditions supported this (as detailed in the following sections of the results). The one participant who did not want to supervise was an overseas trained doctor, and did not feel confident enough at this point in their career to take up supervision. The concern was mainly due to knowledge of Australian training and role as a parent, but it was conceded that this would likely change over time. We decided not to demarcate the results based on supervisor status as the participants largely reflected supervision occurring along a continuum, whereby a common factor was that they reflected about supervision in their context across a breadth of past, present and future experience.

### Quality of clinical care

Most (19) GPs in these rural Tasmanian communities perceived that registrars positively impact the quality of care, both directly: “they usually really want to do a good job and so I think that elevates the standard of care” (#12, > 5 FTE, outer regional), and indirectly through their impact on supervisors and on other practice staff: “It makes you stay up to date; it makes you think about your own practice, it makes me safer, in clinical reasoning and so forth” (#15, < 5 FTE, outer regional). Another participant noted a registrar who was “extremely competent” so “we were sometimes able to ask him [the registrar] about things that we really weren’t certain about” (#17, > 5 FTE, inner regional). The presence of registrars was noted to be “a positive experience for the whole practice not just the other doctors… I think that everybody learns from having a registrar there.” (#16, < 5 FTE, outer regional).

Along with new evidence-based practice, registrars also bring their modern learning styles and technology skills which can benefit the practice: “if I want to explain something about a toe, you know, straightaway we’ve got a 3D image” (#13, < 5 FTE, outer regional). A few participants also noted that registrars can provide opportunities for self-reflection. One participant explained “you … have the ability to reflect upon your own consulting style … when you’re looking closely at someone else’s” (#11,< 5 FTE, outer regional area) and another noted “If you’re critiquing… how other people are doing stuff, you’re also critiquing and analysing how you do stuff” (#13, < 5 FTE, inner regional).

Some (7) participants also commented that in addition to the benefit of extra consulting capacity, registrars can directly benefit patients. One noted “as far as the patients are concerned… they love them and they’re always heart broken when they leave” (#15, < 5 FTE, outer regional). Patient-specific benefits included registrars providing more choice so that patients could see a different doctor if they did not like the other doctors, extra capacity so patients have improved access, that registrars have more time to spend with patients and listen to them, and that seeing a registrar can provide a greater sense of anonymity if patients want to talk about an issue with a doctor they do not already know through other community interactions (e.g. school or sports).

Yet not all registrars bring these benefits. A few (6) participants, across a variety of practice sizes and locations, talked about the challenges of supporting, and working with, a registrar, when the registrars were impacting negatively on quality of care:

it was difficult because we had patients complaining about him… I saw him hurt one patient just using an auriscope and gouging the inside of a guy’s ear by sticking the thing in his ear with poor technique even though I’d taught him the correct technique” (#,1, >5 FTE, inner regional area).

A few others (5) discussed a mismatch between the registrars’ and the practices’ expectations, style of work, location, or culture, such “we work very much on a team model and registrars that don’t work to that kind of mentality – we’ve had a few that haven’t lasted” (#5, > 5 FTE, outer regional area).

### Busy-ness: help or hinder?

Many (17) of the GPs in these rural communities talked about access issues related to how busy their practices were, describing them as: “very busy clinic… [the] doctors who work in our practice … work pretty hard” (#1, > 5 FTE, inner regional area), “…just too busy” (#2, > 5 FTE, outer regional area), and “it’s pretty small, but very, very busy” (#11, < 5 FTE, outer regional area). The impact of taking on a registrar was perceived in two divergent ways – for some practices a registrar could help alleviate the busy-ness, but for other practices taking on a registrar added to the busy-ness. For example, supervising registrars could provide extra workforce capacity, benefitting GPs and their patients with “an extra pair of hands” which assisted in different ways: “getting home at six-thirty instead of eight o’clock” (#1, > 5 FTE, inner regional area), or providing “the chance to have people come in on the day, otherwise … I am booked out for about four weeks in advance” (#24, > 5 FTE, outer regional).

Conversely, trying to support registrars when so busy can mean it is challenging to “juggle the schedule” (#12, > 5 FTE, outer regional). Practice size was a key influence on the ease or difficulty of supervising registrars while maintaining patient access to care. A few (5) participants noted that registrar supervision could be particularly difficult for smaller practices compared to larger practices. For example, managing the teaching load could be difficult compared to larger practices which have more GPs where they can “share the load (#2, > 5 FTE, outer regional). One participant explained:

It might be alright in a bigger practice where you can share it around, and do a bit here and a bit there, but … for me to set aside three hours a week, for a basic [a registrar undertaking their first term of General Practice Training 1 (GPT1)] registrar, is just crazy (#11, <5 FTE, outer regional area).

Other responsibilities that rural GPs carry, such as servicing the local hospital, can similarly impact on the ability to take on registrars by making potential supervisors feel “stretched too thinly” (#17, > 5 FTE, inner regional).

### Patient access

An additional benefit to practices was that the registrar may stay beyond the training, improving patient access in the medium and longer term: “we got her in about 2010, I think, so she went through, got her fellowship, she then stayed with us for another… four to five years” (#11, < 5 FTE, outer regional area). Many (14) participants undertook supervision in the hope of securing a doctor to support their succession planning, but there were few instances where this directly occurred. One participant, considering the seemingly insurmountable difficulties of recruiting doctors to remote Tasmania, reflected “you can’t conscript … I’d like to, but [we’re] not allowed to” (#6, < 5 FTE, remote).

A further issue related to access was what happened when the supply of registrars was inconsistent. Some GPs noted that when a registrar leaves the extra work done by the registrar falls “back on the existing doctors … to see all these additional patients” (#16, < 5 FTE, outer regional) and “the registrar leaves and then all of a sudden we’ve got more patients than we know what to do with again” (#,1, > 5 FTE, inner regional area). The “downside of having intermittent registrars” (#1, > 5 FTE, inner regional area) and the need for an ongoing flow of registrars for consistent provision of GP services in rural towns was further highlighted: “If you were able to guarantee a practice registrars that would… make a massive difference … then you know you can build up your practice [take on new patients] because you’ve got enough doctors” (#15, < 5 FTE, outer regional).

### Energising rural GPs

Participants found supervision activities were enjoyable and supported them to better address community needs. Registrars could also influence a practice through their “youthful energy in the practice” (#12, > 5 FTE, outer regional). One participant reflected that “they’ve got a bit more energy and a bit more enthusiasm… rural doctors, can get crusty and grumpy; it protects you from that a little bit too” (#15, < 5 FTE, outer regional).

Many (15) participants who currently or had previously supervised noted the professional enjoyment and satisfaction they gained from registrar supervision, such as the “fulfilment of knowing that you’re helping with a new generation of doctors” (#13, < 5 FTE < outer regional) and “watching them grow… it’s a satisfying job” (#16, < 5 FTE, outer regional). The enjoyment of teaching was also a particular benefit for some (8). Participants also noted increased personal satisfaction and developing new friendships as rewards for supervising; that “it’s just nice seeing new and different people all the time” (#15, < 5 FTE, outer regional), and that it can be “a lot of fun” (#24, > 5 FTE, outer regional).

GPs initially trained outside of Australia expressed some specific difficulties. One overseas trained GP, who indicated that they did not want to become a supervisor, could still identify positive experiences of their informal co-supervision:

when I was starting here, I’d be very hesitant to answer a registrar’s question, but now …I’ve been here four years, so when they ask me a question they’re similar to the patients I’ve seen before … I find it very fulfilling because you’re able to help someone and pass on your experience to them (#12, >5 FTE, outer regional).

Another participant, who was also an overseas trained doctor and who had previously provided co-supervision, believed their background could support other similar doctors who were undertaking registrar training in Australia: “Australian graduates…will never be able to relate to what somebody who’s coming from outside has experienced, so that’s the one reason that I want to be involved - in making their path a little easier” (#5, > 5 FTE, inner regional).

But for some overseas trained doctors, a lack of confidence may limit their participation in registrar supervision: “my practice principal talked to me about ‘would you like to supervise a registrar’ and I told him I am not confident - I don’t think I can do it” (#21, > 5 FTE, outer regional).

### Business factors

Key to any supervision in the rural context studied, was having a sustainable business model to support it and actually having registrars allocated to a practice. This data needs to be interpreted from the basis that it is a requirement of practices to employ registrars, that is, they cannot be contractors, whereby viable income is essential to cover all employment on-costs.

Concerns around the financial aspects of supervision were common as participants noted supervision “eats in on their ability to earn” (#25, < 5 FTE, outer regional), and “there’s no financial incentive really because registrars and first year registrars don’t tend to make any money for the practice (#2, > 5 FTE, outer regional).

One participant, who had never supervised, but had wanted to and investigated it, explained that:

we decided that having a GP registrar was not going to be cost effective… GP registrars are employees so … if a GP registrar was unwell and could not work suddenly, I would still have to bring in an income to cover that registrar’s leave (#10, < 5 FTE, inner regional).

For this participant to start supervising it would be an “issue of getting the financials right”.

The challenges of bringing in income when being part-time can also impact on intention to supervise or not: “because I’m only working part-time, from a financial point of view it’s also difficult because … I need to see patients to make money and … supervising a registrar slows that down a little bit” (#16, < 5 FTE, outer regional).

Stage of career may be another influence on how important the business aspects of supervision are, with one participant in the later stage of their career seeming less concerned by this despite acknowledging the poor remuneration: “you do get paid a little bit but you know it certainly doesn’t make up for cutting back on how many patients we see, but to me that’s not, at this stage, all that important” (#12, > 5 FTE, outer regional). Stage of life may also impact decisions: for one participant who was an accredited supervisor and currently providing co-supervision the “time pressures over the year with a growing family” meant that the participant would not commit to be a main supervisor role at this point in time.

GP supervisors were also cognisant that tensions could also arise when a mismatch emerged between the wages paid to registrars and the income they generated through fee-for service consultations: “They [the registrar] just turned out to be really average and a lot of hard work, and could never get past about two patients an hour” (#11, < 5 FTE, outer regional area).

Visiting medical officer services provided to the local hospital needed to be considered within the overall financial models: “If we could convince the hospital of the value of having registrars and find some sort of pay structure that would help” (#15, < 5 FTE, outer regional). Another participant thought that the financial models for registrar supervision should be changed in other ways: “The way practices are remunerated, and the way registrars are paid is something that… needs looking at, because initially they are seeing few patients and are not charging enough, so practices lose money (#2, > 5 FTE, outer regional).

Some rural GPs wanted to supervise but found they were not able to access a registrar. Participants indicated they wanted a fairer process for registrar allocation, such as “some sort of process that equitably distributes registrars between city and regional centres” (#1, > 5 FTE, inner regional area) or “I reckon they should just bloody force them out (#11, < 5 FTE, outer regional area).

The uncertainty involved in 6–12 monthly rotational allocations negatively affected business planning, particularly if practices were only sometimes receiving registrars. As noted earlier when a registrar leaves it creates problems as the practice can then have more patients than they can effectively service, with the responsibility for the care of these additional patients falling to the remaining doctors. For rural GPs “continuity would really help. If we knew we were going to get a registrar year in year out, even if they were different levels [of experience], that would help” (#15, < 5 FTE, outer regional).

Having the physical space to take on a registrar can also impact the decision to take on a registrar and a registrar’s experiences: “we, at times, have problems with rooms, and I think if you’re going to be a registrar you shouldn’t be shunted from room to room, you need a dedicated room” (#11,< 5 FTE, outer regional area).

## Discussion

This study provides, for the first time, an in-depth understanding of the experience of GPs participating in registrar supervision in small rural communities. It particularly provides insights into supervision in the context of GPs working in smaller rural towns (< 25,0000 people) outside of major regional centres (Hobart and Launceston in this study). GPs supervising denoted positive impacts of supervising on quality of clinical care, various aspects of the busy-ness of the practice and improving patient access to services. Rural GPs in this study were energised by supervising but a diverse range of business factors were identified in relation to supervising and supervision systems, which are potentially important for informing how to expand rural GP training as an issue for training decentralisation. While quantitative data has already identified that over half of rural GPs supervise, not related to town size [11], other evidence suggests a need to increase GP supervisory capacity in underserved rural and remote areas where primary care needs are higher [12]. Our study specifically provides information which helps to understand how the expansion of more rural GP training could be facilitated. Firstly, by promoting the benefits for rural, not just regional GP supervising, but secondly by improving the systems which have the potential to impact business factors for GPs in rural towns. On balance, the positive aspects of participating in supervising registrars relate to improving the GP’s work satisfaction/energy, assisting with clinical quality and improving service access by the rural community. However, for GP supervisors based more rurally than major regional centres being able to realise the gains of supervision is precarious and dependent on registrars nominating to train away from regional centres. This is because the current AGPT training policy requiring distribution of 50% of registrars to a rural pathway does not differentiate cities from rural areas in Tasmania (all of Tasmania is considered MMM2–7). It is possible that this could be addressed through more nuanced guidelines of the RTO in Tasmania, or perhaps our findings could prompt a review of the national ‘rural pathway’ policy, for example by embedding some rurality quotas.

The impact of business factors for GPs supervising in rural areas (outside of regional centres) provides a new critical lens on how decisions by policy-makers (e.g. Department of Health, Colleges and RTOs), such as whether a practice gets a registrar, the continuity of the registrars (where possible minimising short-term rotations training for its implications for practices in small rural communities) and registrar stage of training (clinical competence), might impact rural GPs already managing enormous demands for patient primary care. Registrars who have progressed further through their training may bolster practice sustainability by being able to boost independent workforce capacity, easing workload burden in the short term, but longer-term, the rotational training system also presents difficulties related to balancing competing demands on GP supervisors’ time, infrastructure, patient demands and remuneration. The policy and educational rationale of securing a more constant supply of rotating registrars has recently been re-visited in Australia so that registrars are no longer required to move practice every 6 months; our findings reinforce this policy change, given that this study identified that six monthly rotation is a burden and deterrent for hosting rural practices. Overall, the finding that business factors are a key driver fits with O’Sullivan et al.’s [11] research, finding that GP practice factors, such as practice size, rather than rurality, were associated with supervision participation. Aligned with these business issues is research showing greater financial challenges of teaching in rural versus urban Australian general practices, with rural practices experiencing a financial loss for GP registrar teaching compared with urban practices which made a small financial gain [21]. The need for improved remuneration of GP supervisors has also been raised by others, not specific to rural GPs [6, 22].

The commitment to supervision, and the enjoyment that the GPs gained from it is consistent with other research and may not be unique to rural GPs [13, 23–26]. However, for rural GPs working in more isolated conditions, such impacts may play out to improve job satisfaction and retention, in a way that they do not impact metropolitan providers.

Overall, registrars could also provide an important informal component of professional development for rural GPs who typically find it difficult to access face-to-face professional development activities which are often held in metropolitan locations. Clinical education is generally discussed as a one-way learning processes [27, 28] with the registrar learning from the GP supervisor and the clinical experiences gained under supervision. However, our data show that supervising registrars provides very beneficial two-way learning opportunities for both the registrar and for the supervising GP, and also provides learning opportunities for other practice staff. Supervision opportunities are a unique chance to engage in reciprocal learning and professional development, facilitated by the interactions and interconnections between networks of people [29].

Our findings also identified several other issues: that there are GPs providing unaccredited, ad hoc “corridor” supervision without formal accountability for registrar learning. These GPs enjoy these experiences, though they may not easily be drawn into models that take more time and responsibility. Also, while registrar supervision is traditionally considered a master/apprentice model, with other supports (such as medical educators and supervisor liaison) our findings suggest that this model may be problematic in its intensity for GPs in small towns. GPs working in more isolated areas, in early career may not feel ready nor have the time to take on the role of ‘master’. Also overseas-trained doctors enjoyed supervision and their contribution to helping other doctors from overseas to orientate and learn to be Australian GPs. However, this group faces unique challenges, notably they may need additional encouragement, mentorship and a strong sense of their importance to the learning systems to formally take on supervision roles. Systematising alternate models for GPs in small rural towns may assist, giving confidence and dedicated capacity for supervision roles. Alternative models of supervision, such as blended [30] or remote [31], or taking on part-time registrars [32] might encourage GPs at different career stages or with different backgrounds to participate as supervisors.

We only recruited GPs from Tasmania, which may be considered a limitation, and this study design could be repeated in other contexts, although we contend that the main drivers of the issues for the GPs working outside of major regional centres of Hobart and Launceston in Tasmania could be relevant to other small rural locations within Australia and elsewhere.

## Conclusions

Our study provides unique new evidence that GPs in small rural communities (outside of regional centres) perceive enormous gains from supervising registrars for improving quality of medical care, improving access to primary care for the community and energising their ongoing work as a rural GP. Despite their interest, a range of business factors have the potential to impact supervision in small rural communities, such as certainty about getting a registrar, continuity of registrar supply and practice income as affected by registrar competence. Decentralising GP education depends on increasing supervision capacity in rural areas, itself requiring supervision policies and systems which advocate of the benefits of supervising/learning in this context and promoting more viable supervision models tailored to the business needs of rural GPs and their practice context.

## Supplementary information

**Additional file 1.**

## Data Availability

The datasets used and/or analysed during the current study are available from the corresponding author on reasonable request and subject to approval by Monash University Ethics.

## Abbreviations

AGPT: Australian General Practice Training Program
ASGS-RA: Australian Statistical Geography Standard-Remoteness Area
FTE: Full-time equivalent
GP: General practitioner
GPT: General Practice Training
MMM: Modified Monash Model
RTOs: Regional Training Organisations

## References

[1] Walters LK, McGrail MR, Carson DB, O'Sullivan BG, Russell DJ, Strasser RP (2017). Where to next for rural general practice policy and research in Australia?. Med J Aust.

[2] Australian Government (2019). General practice training in Australia - the guide.

[3] Humphreys JS, Jones JA, Jones MP, Mildenhall D, Mara PR, Chater B (2003). The influence of geographical location on the complexity of rural general practice activities. Med J Aust.

[4] McGrail M, Russell D, Campbell D (2016). Vocational training of general practitioners in rural locations is critical for Australian rural medical workforce supply. Med J Aust.

[5] Trumble SC (2011). The evolution of general practice training in Australia. Med J Aust.

[6] Thomson J, Anderson K, Mara P, Stevenson A (2011). Supervision — growing and building a sustainable general practice supervisor system. Med J Aust.

[7] Sen Gupta T, Reeve C, Larkins S, Hays R. Producing a general practice workforce: Let’s count what counts. Aust J Gen Pract. 2018;47(8):514–7. 10.31128/AJGP-02-18-4488 30114889

[8] Hendrie D (2019). General practice training applications have dipped: What can be done?.

[9] Playford D, May JA, Ngo H, Puddey IB (2020). Decline in new medical graduates registered as general practitioners. Med J Aust.

[10] O'Sullivan BG, McGrail MR (2020). Effective dimensions of rural undergraduate training and the value of training policies for encouraging rural work. Med Educ.

[11] O'Sullivan B, Russell D, McGrail M, Sampson M, Warrington A, Wallace G (2019). Factors related to rural general practitioners supervising general practice registrars in Australia:'A national cross-sectional study'. Aust J Gen Pract..

[12] McGrail M, Russell D, O’Sullivan B, Reeve C, Gasser L, Campbell D (2018). Demonstrating a new approach to planning and monitoring rural medical training distribution to meet population need in north West Queensland. BMC Health Serv Res.

[13] Ingham G, Fry J, O'Meara P, Tourle V (2015). Why and how do general practitioners teach? An exploration of the motivations and experiences of rural Australian general practitioner supervisors. BMC Med Educ.

[14] Young L, Peel R, O’Sullivan B, Reeve C (2019). Building general practice training capacity in rural and remote Australia with underserved primary care services: a qualitative investigation. BMC Health Serv Res.

[15] Teherani A, Martimianakis T, Stenfors-Hayes T, Wadhwa A, Varpio L (2015). Choosing a qualitative research approach. J Grad Med Educ.

[16] Australian Bureau of Statistics (2018). 3101.0 - Australian Demographic Statistics, Dec 2017.

[17] Denzin NK, Lincoln YS (2000). The Handbook of Qualitative Research.

[18] Etikan I, Musa SA, Alkassim RS (2016). Comparison of convenience sampling and purposive sampling. Am J Theor Appl Stat.

[19] Braun V, Clarke V (2006). Using thematic analysis in psychology. Qual Res Psychol.

[20] Strauss AL, Corbin JM (1998). Basics of qualitative research: techniques and procedures for developing grounded theory.

[21] Laurence C, Black L, Karnon J, Briggs N (2010). To teach or not to teach? A cost–benefit analysis of teaching in private general practice. Med J Aust.

[22] Thistlethwaite JE (2006). Altruism can no longer support community-based training. Med J Aust.

[23] Ingham G, O'Meara P, Fry J, Crothers N (2014). GP supervisors – an investigation into their motivations and teaching activities. Aust Fam Physician.

[24] Radford J (2017). A survey of strategies for increasing the number of medical learners in all Tasmanian general practices. Focus Health Prof Educ: A Multi-Disciplinary J.

[25] McCallum M, MacDonald S, McKay J. GP speciality training in areas of deprivation: factors influencing engagement. A qualitative study. BJGP Open. 2019;3(2). 10.3399/bjgpopen19X101644. 10.3399/bjgpopen19X101644 PMC6662869 31366675

[26] Garth B, Kirby C, Nestel D, Brown J (2019). Your head can literally be spinning': a qualitative study of general practice supervisors' professional identity. Aust J Gen Pract.

[27] Carrington G (2004). Supervision as a reciprocal learning process. Educ Psychol Pract.

[28] Waters L, Lo K, Maloney S (2018). What impact do students have on clinical educators and the way they practise?. Adv Health Sci Educ.

[29] Jörg T (2009). Thinking in complexity about learning and education: a programmatic view. Complicity: An Int J Complexity Educ.

[30] Ingham G, Fry J (2016). A blended supervision model in Australian general practice training. Aust Fam Physician.

[31] Wearne SM, Teunissen PW, Dornan T, Skinner T (2015). Physical isolation with virtual support: registrars’ learning via remote supervision. Med Teach.

[32] Khan N, Usherwood T (2019). ‘We are not invincible’: a qualitative study of self-care practices by Australian general practice registrars. Aust J Prim Health.

