# Supplementary material for: An exploration of the experiences of GP registrar supervisors in small rural communities: a qualitative study
Source: BMC Health Serv Res. 2020 Sep 5;20:834. doi: 10.1186/s12913-020-05697-2 (PMC7487663; doi:10.1186/s12913-020-05697-2)
Supplement: Supplementary file 1 — Additional file 1. [file 12913_2020_5697_MOESM1_ESM.docx]

**Schedule of questions for the semi-structured interviews**

**Supervising GP Registrars – who does and doesn’t participate and why?**

INTERVIEWER

- **Thanks for agreeing to do this interview. It is 45 minutes only and is being recorded so it can be transcribed and the data can be most effectively analysed. But your information will be anonymous and only reported in a summarised form.**
- **The interview has 3 sections**
- **If you want to stop or have a break at any time please let me know.**
- **To manage the time, I will give you a warning when we have 20 minutes left.**
- **I will also let you know when we have 5 minutes left.**

**Interview ID** [the GP has been allocated a random number from [name], which will be used as the ID for each interview]

Section 1

**Firstly I want to ask you some short questions about you and your practice.**

| **Question** | **Answer (circle and write text)** |
| --- | --- |
| **A. About you** |  |
| 1. When did you complete your basic medical degree?   In Australia or overseas? | Year:  Australia  Overseas |
| 1. When did you start working as a GP?   *[When finished vocational training or commenced employment in general practice for those who did not undertake vocational training?]* | Year: |
| 1. How long have you been working as a GP for?   And in total, how many years have you worked in rural general practice? | Years been GP_________  Rural practice years___________ |
| 1. Are you vocationally registered? If so, with which College? And were you grandfathered? | No  Yes RACGP, Yes / No Grandfathered  Yes ACRRM, Yes/ No Grandfathered |
| **B. About the practice** |  |
| 1. Where is your main practice located? | Town and postcode |
| 1. How long have you worked in this practice for? | Years______ |
| 1. How many other GPs and other staff are in the practice/s with you? | < 5 GPs  5+ GPs  Number other staff (practice manager, allied health, receptions staff)__________ |
| 1. Is the practice currently accredited as a GP training post? | No  Yes |
| 1. How many sessions do you undertake in an average week? | Sessions in average week |
| 1. Do you also work in a hospital? If yes, what percentage of your total practice time in an average week is spent there? | No  Yes, % practice time in average week |
| 1. Do you work in other settings as well (e.g. ACCHO, community health)? | No  Yes, % total practice time |
| 1. Do have other medical qualifications– e.g. in areas like obstetrics, anaesthetics or other? | No  Yes, which areas? |
| 1. Do you currently practice in these areas? | No  Yes, which ones |

Section 2:

**Now can you tell me a bit about your experience with supervising GP registrars**

| **Question** | **Answer** |
| --- | --- |
| **A. EVER supervised** |  |
| 1. Have you ever supervised GP registrars? If so, for how long? | No **Go to Q16**  Yes, years_______ **Go to Q15** |
| 1. How many registrars do you typically supervise or participate in supervising in any given year? | Number_______________ |
| **B. CURRENT supervising** |  |
| 1. Are you currently supervising GP registrars? | No **Go to Q20**  Yes **Go to Q17** |
| 1. How many and at what stage of training? | Registrars in year 1- number____________  Registrars in year 2 - number____________  Registrars in year 3 - number____________ |
| 1. What role do you play - are you the lead or co-supervisor to these registrars? | Role:  Number you **Lead**  Number you **co-supervise**  Other role, specify |
| 1. Are you expected to supervise registrars as part of working in your current practice? | Part of working in this practice  I am allowed to decide to participate  Both part of practice and I can decide |
| 1. Are you currently supervising medical students? | No – **Go to section 3**  Yes – **Go to Q21** |
| 1. Do you typically supervise medical student and GP registrars concurrently in the practice? Why? Why not? | No  Yes |

Section 3: **So can I confirm before preceding to the Section 3, you have:**

**A Never supervised GP registrars**

**B Supervised GP registrars in the past**

**C Are currently supervising GP registrars**

**D Exclusively currently supervise medical students only**

**Now I am going to ask questions about the reasons why?**

**ASK ONLY THE QUESTIONS ACCORDING TO GROUPING ABOVE, either A, B, C or D**

| **Question** | **Answer** |
| --- | --- |
| **A Never supervised GP registrars** |  |
| 1. Can you tell me about why **you have never** supervised GP registrars? |  |
| 1. What do you think would be some of the positive aspects of supervision for you? For the practice? | You?  The practice? |
| 1. What do you think would be some of the challenging aspects of supervision for you? For the practice? | You?  The practice? |
| 1. Would you like to supervise registrars in the future? | No, why  Yes, why |
| 1. What would help **you** to become a supervisor? |  |
| 1. What support **from the practice** would help you to become a supervisor? |  |
| **B Supervised GP registrars in the past** |  |
| 1. You’ve **previously supervised GP registrars, but you are not currently supervising**. Can you tell me how you found supervising in the past? |  |
| 1. What is the main reason you **did not** continue to supervise? | Related to you?  Related to the practice? |
| 1. What were some of the positive aspects of supervising for you? For the practice? | You?  The practice? |
| 1. What were some of the challenging aspects of supervising for you? For the practice? | You?  The practice? |
| 1. Would you like to supervise registrars again in the future? | No, why  Yes, why |
| 1. What would help **you** to supervise again? |  |
| 1. What support **from the practice** would help you to supervise again? |  |
| **C Currently supervising GP registrars** |  |
| 1. So you **currently supervise registrars**? Can you tell me how you find this experience? |  |
| 1. What is the main reason that **you participate in** supervising? | Related to you?  Related to the practice? |
| 1. What are some of the positive aspects of supervising? For you? For the practice? | You?  The practice? |
| 1. What are some of the challenges of supervising for you? For the practice? | You?  The practice? |
| 1. How do you find the supervision workload with respect to managing your normal clinical load? | Low  Medium  High |
| 1. Do you plan to continue supervising? | No, why  Yes, why |
| 1. Is there anything that would make supervising more sustainable going forward? | For you?  The practice? |
| **D Exclusively supervising medical students** |  |
| 1. So you **only supervise medical students** and you haven’t ever supervised registrars. What is the main reason you supervise medical students? |  |
| 1. Would you like to supervise GP registrars in the future? |  |
| 1. What would help **you** to start supervising registrars? |  |
| 1. What support **from the practice** would help you to supervise registrars? |  |
| 1. If you took up GP registrar supervision would you continue to supervise medical students? | No, why  Yes, why |

- **We have reached the end of the interview.**
- **Thank you for taking the time out of your busy schedule to participate.**
- **I will notify [name] that the interview has been completed and she will be in touch with the gift voucher.**
- **We expect the results will be available via a communique from GPTT and GPSA.**
- **Please keep a copy of the explanatory statement and feel free to contact [name] at any time to find out more information about the study.**
